# Design and Characterization of Ring-Curve Fractal-Maze Acoustic Metamaterials for Deep-Subwavelength Broadband Sound Insulation

**DOI:** 10.3390/ma18153616

**Published:** 2025-07-31

**Authors:** Jing Wang, Yumeng Sun, Yongfu Wang, Ying Li, Xiaojiao Gu

**Affiliations:** 1 Academy of Fine Art and Design, Xuchang University, Bayi Road 88, Xuchang 461000, China; 2 School of Mechanical Engineering and Automation, Northeastern University, Shenyang 110819, China; sunyumengss@163.com; 3 School of Mechanical Engineering, Shenyang Ligong University, Nanping Middle Road 6, Shenyang 110159, China; liying8282@sylu.edu.cn (Y.L.); 18804033860@163.com (X.G.)

**Keywords:** curve fractal channels, acoustic metamaterials, deep subwavelength, broadband bandgaps, transmission loss

## Abstract

Addressing the challenges of bulky, low-efficiency sound-insulation materials at low frequencies, this work proposes an acoustic metamaterial based on curve fractal channels. Each unit cell comprises a concentric circular-ring channel recursively iterated: as the fractal order increases, the channel path length grows exponentially, enabling outstanding sound-insulation performance within a deep-subwavelength thickness. Finite-element and transfer-matrix analyses show that increasing the fractal order from one to three raises the number of bandgaps from three to five and expands total stop-band coverage from 17% to over 40% within a deep-subwavelength thickness. Four-microphone impedance-tube measurements on the third-order sample validate a peak transmission loss of 75 dB at 495 Hz, in excellent agreement with simulations. Compared to conventional zigzag and Hilbert-maze designs, this curve fractal architecture delivers enhanced low-frequency broadband insulation, structural lightweighting, and ease of fabrication, making it a promising solution for noise control in machine rooms, ducting systems, and traffic environments. The method proposed in this paper can be applied to noise reduction of transmission parts for ceramic automation production.

## 1. Introduction

Low-frequency noise is difficult to attenuate under the constraints of the mass law, and conventional sound-insulation strategies typically require bulky materials that prove ineffective in space- or weight-limited applications [1]. Acoustic metamaterials have overcome this limitation by introducing subwavelength local resonances or artificial slow-wave channels, enabling pronounced sound attenuation at thicknesses far below the wavelength [2]. Previous work has demonstrated that both locally resonant units [3,4,5,6,7] (e.g., membrane–plate, Helmholtz, and Mie resonators) and maze-type metamaterials [8,9,10,11] can produce bandgaps in targeted frequency ranges; however, the former often operate only near a single resonance frequency, yielding limited bandwidth, whereas the latter—though capable of generating multiple gaps via folded propagation paths—commonly employ straight-cornered channels whose effective path length is constrained by sharp angles and channel widths, thus imposing bottlenecks on refractive index and insulation performance. Since the pioneering demonstration of locally resonant acoustic metamaterials by Liu et al. [12], researchers have exploited subwavelength artificial structures to manipulate low-frequency sound waves. Representative designs include arrays of rubber-coated metal cylinders achieving single-negative density or bulk modulus, tensioned-membrane metamaterials with negative dynamic mass, and Helmholtz-cavity or Mie-resonance metasurfaces realizing ultra-high or zero reflection. Nonetheless, these structures typically deliver insulation enhancement only at one or a few narrow resonant bands, falling short of broadband noise-suppression requirements.

To extend bandgap bandwidth, two main approaches have emerged. One is to couple multiple local resonances within a single unit to create “multi-frequency” gaps; the other is the “coiling-up-space” strategy, which dramatically lengthens the effective acoustic path to achieve ultra-low phase velocity and high refractive index, thereby forming multiple Bragg–local hybrid gaps at subwavelength thicknesses. Key examples include Ren et al. [13], who introduced an impedance-matched coiled-cavity absorber that, through coupled multi-void channels, achieved a 4.55× bandwidth enhancement at 602 Hz under a λ/10.96 thickness, with impedance-matching–induced dissipation producing dual perfect-absorption peaks. Liu et al. [14] proposed coiled-space metamaterials that coordinate monopole and dipole resonances to dissipate 99% of incident sound at two characteristic frequencies, thereby widening the blocking band between these resonances. Li et al. [15], who developed hierarchical maze metamaterials and, via finite-element and Bloch-theorem analyses, elucidated graded-structure bandgap tuning and wave-directionality mechanisms, achieving synergistic low-frequency gap expansion and vibration attenuation through local-resonance–gradient coupling. Liang et al. [16] established a subwavelength Dirac-cone tuning paradigm in a triangular maze, shifting topologically protected edge-state frequencies down to 684 Hz and leveraging scatterer-rotation-induced valley Chern-number transitions for robust low-frequency control. Huang et al. [8], who formulated a resonator-number–spacing co-design and, using a dimer configuration, realized full-aperture sound transmission with a pressure-gradient control mechanism that improved isolation efficiency by 40%, offering a parameterized framework for integrated acoustic-device design.

Despite these advances, existing maze-type metamaterials predominantly use right-angled or sharp-corner geometries; scattering at sharp corners and fabrication-precision limits constrain minimal channel width and iteration order, thereby restricting the effective path length and the number of achievable bandgaps. Moreover, the abrupt curvature changes inherent to zigzag structures introduce additional viscous losses and increase manufacturing difficulty.

Self-similar fractal acoustic metamaterials, by integrating the unique advantages of fractal geometry, offer new avenues for sound-wave manipulation—especially in low-frequency broadband control and lightweight design [17,18,19,20,21,22]. He et al. [23] introduced a subwavelength two-dimensional fractal metamaterial based on Hilbert-curve topology and spatial vorticity, achieving switchable near-zero/negative density and enabling unidirectional sound transmission with negative refraction at subwavelength scales. Xia et al. [24] proposed a fractal coiling strategy in which self-similar topological iterations enhanced bandgap depth by 40%; the fractal-induced local-field amplification surpassed conventional Bragg-scattering limits, producing deep subwavelength gaps and establishing a “geometric-fractal × topology-optimization” paradigm for miniaturized acoustic filters. Liu et al. [25] demonstrated that fractal-order tuning in coiled-space metamaterials significantly broadens broadband insulation along the X-axis via band-folding effects, yielding continuous stopbands at deep-subwavelength thicknesses. Cao et al. [26] realized fractal-maze metamaterials using recursive triangular topologies coupled with thermo-viscous dissipation, reaching a peak absorption coefficient of 0.99 in the 50–400 Hz range. Zhang et al. [27] developed fractal honeycomb mechanical metamaterials and, through mixed analytical–numerical validation, revealed how fractal ratio and concave-corner geometry shift bandgaps down to 80–400 Hz, with second-order fractals producing the lowest frequency gaps.

In summary, existing research in the field of low-frequency acoustic metamaterials has made significant strides, covering local-resonance designs, coiling-up-space strategies, and fractal-structure approaches. Self-similar fractal metamaterials, in particular, have garnered attention for their scalability and subwavelength control capabilities. However, most current fractal designs rely on sharp-cornered or straight-channel geometries (e.g., Hilbert curves or triangular fractals), which extend the effective acoustic path and broaden bandgaps—inevitably introducing corner scattering, extra viscous dissipation, and manufacturing complexity, thus limiting further gains in refractive index and sound-insulation performance. Moreover, although some works have achieved breakthroughs in bandgap expansion and multi-resonance behavior, there remains a lack of systematic exploration of novel fractal-channel architectures featuring continuous curvature and recursive constructability. Therefore, designing acoustic metamaterials with smooth, nested curved fractal channels promises to avoid corner losses and fabrication challenges while achieving exponential path-length growth, enabling wider, deeper, and lower-frequency bandgap control within thinner structures. In this paper, we proposed a ring-curve fractal-maze metamaterial and will use numerical simulations and experimental measurements to comprehensively assess its bandgap evolution, effective parameter behavior, and transmission-loss performance. Our goal is to overcome current bottlenecks in low-frequency broadband insulation efficiency, manufacturability, and tunability of maze-type metamaterials and to provide theoretical foundations and design guidelines for next-generation lightweight, high-performance, and engineering-compatible low-frequency sound-insulation structures.

The paper is organized as follows. Section 2 describes the geometric design and analytical modeling of the curve fractal unit. Section 3 presents numerical band structure and negative-parameter retrieval results. Section 4 analyzes transmission loss and acoustic-pressure distributions. Section 5 discusses sensitivity to operating conditions and potential applications. Finally, conclusions are summarized and future research directions are outlined.

## 2. Proposed Method

### 2.1. Geometric Recursion Rule

**Figure 1:**
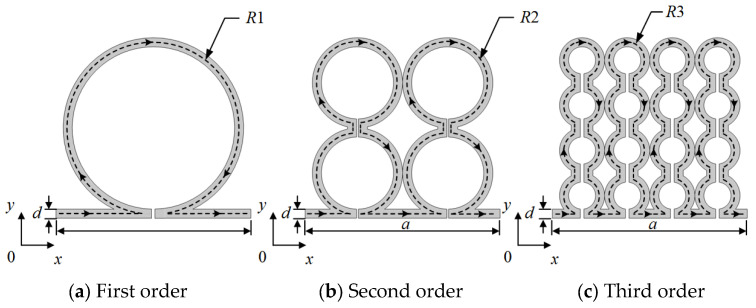
(**a**–**c**) Unit cells of the first- to third-order fractal acoustic metamaterials.

In this work, a fractal structure is reconfigured into a series of coaxial circular tunnels, achieving exponential gains in effective acoustic path length while maintaining minimal geometric complexity. An analytical model is established to relate the fractal order and tunnel width to the effective sound speed, refractive index, and attenuation. The schematic of the proposed ring-type fractal acoustic metamaterial is shown in Figure 1.

Figure 1a–c illustrates three recursive unit cells, each composed of concentric circular tunnels. A single ring is taken as the first-order unit (n = 1). At each subsequent iteration, one sub-ring of radius *R_n_*_−1_ is bisected along two orthogonal directions (horizontal and vertical) into four equal segments, and is replaced by four concentric rings of radius *R*_*n*_ = *R_n_*_−1_/2, while the lattice constant *a* (*a* = 56 mm) remains unchanged. Accordingly, the total number of rings in the nth-order unit is given by the following formula:

(1) $$N_{n}=4^{n-1}(n\geq 1)$$

and the smallest sub-ring radius is calculated as follows:

(2) $$R_{n}=\frac{R_{1}}{2^{n-1}}$$

Because the geometry is perfectly self-similar, the internal tortuosity (or “folding index”) grows exponentially with fractal order, whereas the outer dimension *a* and the inlet/outlet interface remain fixed. This yields a multi-layered, scale-controllable channel network for the subsequent acoustic modeling.

**Figure 2:**
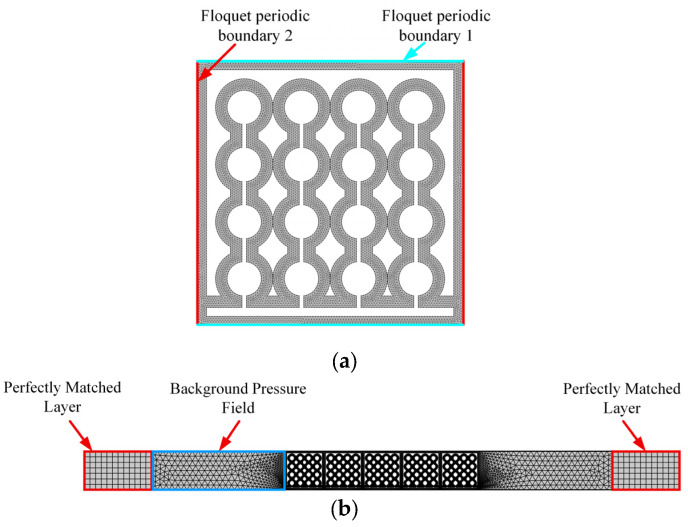
Model settings: (**a**) bandgap calculation model; (**b**) TL calculation model.

A numerical model was established in the pressure acoustics module of COMSOL Multiphysics 6.0. For the bandgap calculation, as shown in Figure 2a, two pairs of Floquet periodic boundary conditions were applied to compute the eigenfrequencies and extract the bandgap. The triangular mesh was used, comprising 9629 mesh nodes and 16,654 triangular elements. For the TL model, as shown in Figure 2b, five phononic crystal units were arranged periodically, with perfectly matched layers at both ends to eliminate acoustic reflections. The region to the left of the phononic crystal was assigned a background pressure field of 1 Pa. This model comprised 19,381 mesh nodes, 31,656 triangular elements, and 168 quadrilateral elements.

### 2.2. Maze Path Length

Acoustic waves propagate along the serpentine path through each concentric ring and couple instantaneously across narrow slits of width *d* (*d* = 2.5 mm). Neglecting the center-to-center spacing between adjacent sub-rings, the equivalent propagation length of the *n*th-order unit can be approximated as follows:

(3) $$L_{eff,n}≃\sum_{k=1}^{n}N_{k}\pi R_{k}=\pi R_{1}\sum_{k=1}^{n}\frac{4^{k-1}}{2^{k-1}}=\pi R_{1}2^{n-1}$$

Here, each increment in fractal order doubles the acoustic path length; by the third order, the path is 2^2^ = 4 times that of the first-order unit, thereby greatly enhancing the phase accumulation within the cell. Equation (3) shows that, owing to the circular (rather than straight) channel geometry, *L_eff_* grows exponentially with base 2, multiplied by the geometric factor *π*.

### 2.3. Order-Dependent Sound Speed and Refractive Index

Let *c*_0_ be the sound speed in the background medium and *a* the lattice constant. The average phase velocity within the *n*th-order unit is then written as follows:

(4) $$c_{eff,n}=c_{0}\frac{a}{L_{eff,n}}=\frac{c_{0}a}{2^{n-1}\pi R_{1}}$$

and the corresponding effective refractive index becomes the following:

(5) $$n_{r,n}=\frac{c_{0}}{c_{eff,n}}=2^{n-1}\frac{\pi R_{1}}{a}$$

Both *c*_eff,*n*_ and *n*_r,n_ follow exponential scaling: increasing *n* by one roughly halves *c*_eff_ and doubles *n*_r_. Consequently, the center frequency of the first bandgap *f*_1,*n*_∝*c*_eff,n_ also decreases in tandem, enabling rapid downshifting of the sound-insulation cutoff frequency under a constant unit size.

### 2.4. Effect of Slit Width D on Acoustic Performance

Although when d ≪ a, the slit width has a negligible effect on the effective propagation length *L*_eff,n_ reducing *d* introduces additional dissipation in the viscous–thermal boundary layer. Hence, the attenuation coefficient can be approximated as follows [28]:

(6) $$\alpha (d)\approx K\sqrt{\frac{\omega \rho_{0}}{2\mu }\frac{1}{d}}$$

where *α*(*d*) is the attenuation coefficient (Np/m), describing the exponential decay rate of sound-pressure amplitude along the propagation direction; *K* is a dimensionless constant accounting for channel geometry and thermo-viscous boundary-layer corrections; *ω* is the angular frequency (rad/s); *ρ*_0_ is the static density of air (kg/m^3^); *μ* is the dynamic viscosity of air (Pa·s).

From (6), $\alpha (d)\propto d^{-1/2}$, meaning the narrower the channel, the stronger the attenuation. Accordingly, the transmission efficiency of the *n*th-order channel is as follows:

(7) $$\eta_{n}=\exp(-\alpha (d)L_{eff,n})$$

which represents the ratio of transmitted to incident acoustic power, and is related to the transmission loss (*TL*) is defined as follows:

(8) $$TL=-10\mathrm{lg}(\eta_{n})$$

Therefore, reducing *d* deepens the bandgap and raises the peak *TL*; however, excessively narrow slits markedly increase viscous–thermal dissipation and manufacturing difficulty. Empirical design guidelines suggest 0.02*a* ≤ *d* ≤ 0.08*a* [29] Conversely, at a fixed fractal order *n*, increasing d reduces viscous damping, slightly up-shifts the local resonance frequency, and weakens refractive-index enhancement. Thus, an optimal balance must be struck among fabrication constraints, bandgap depth, and energy loss.

## 3. Band Structures of the Circular-Loop Fractal Acoustic Metamaterials

### 3.1. Governing Equations and Boundary Conditions

Within each concentric-ring maze channel, air is treated as an inviscid, incompressible ideal fluid, and its harmonic pressure field *p*(*r*)e^jωt^ satisfies the Helmholtz equation, written as follows:

(9) $$\nabla \cdot (\frac{1}{\rho_{0}}\nabla p)-\frac{\omega^{2}}{\rho_{0}c_{0}^{2}}p=0$$

where *ρ*_0_ and *c*_0_ are the density and sound speed of air, respectively. The maze walls are assumed rigid, so a sound-hard (Neumann) boundary condition is imposed on the air–solid interface:

(10) $$\frac{\partial p}{\partial n}=0$$

Ensuring zero normal particle velocity. To simulate an infinite periodic arrangement within a finite computational domain, Floquet–Bloch periodicity is applied on opposite unit-cell boundaries, written as follows:

(11) $$p(r+a)=p(r)e^{-jka}$$

where *a* is the lattice translation vector and *k* the two-dimensional Bloch wavevector.

**Figure 3:**
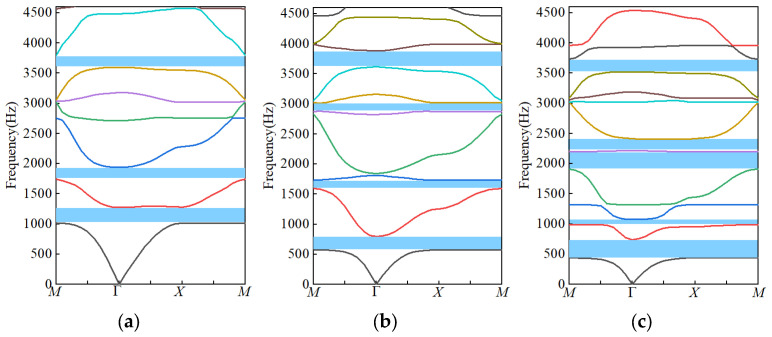
Band structures of fractal acoustic metamaterials at [0 Hz, 4500 Hz]: (**a**) The first-order fractal acoustic metamaterials. (**b**) The second-order fractal acoustic metamaterials. (**c**) The third-order fractal acoustic metamaterials.

Eigenfrequency analysis was carried out in COMSOL Multiphysics. First-, second-, and third-order unit cells were discretized using a fine mapped mesh (minimum element size < 0.02*d*). Eigenfrequencies were extracted pointwise along the high-symmetry path Γ–X–M–Γ in the Brillouin zone, which spans all symmetry-inequivalent directions of the square-lattice irreducible Brillouin zone. The resulting *ω*–*k* dispersion relations are presented in Figure 3.

From Figure 3, the first-order dispersion curve exhibits three primary bandgaps: BG_1_ [1017–1274 Hz], BG_2_ [1742–1939 Hz], and BG_3_ [3600–3790 Hz]. These gaps arise from the coupling of quarter-wavelength local resonances in the rings with Bragg scattering at the lattice scale *a*, yielding a total coverage of approximately 17%. In the second-order band structure (Figure 2b), the maze path length doubles and the effective sound speed halves, shifting the gaps to lower frequencies and increasing their number to four: BG_1_ [577–800 Hz], BG_2_ [1150–1300 Hz], BG_3_ [2875–3020 Hz], and BG_4_ [3614–3890 Hz], with an overall bandgap width of about 25%. The two lower gaps are dominated by λ/4 resonance of the second-order ring (radius *R*_2_), whereas the higher-frequency gaps result from multi-scattering coupling between first- and second-order rings. In the third-order band structure (Figure 3c), the unit cell contains 16 sub-rings. Five distinct bandgaps appear, with center frequencies at 490 Hz, 1050 Hz, 2000 Hz, 2300 Hz, and 3700 Hz—corresponding to normalized frequencies of [0.074, 0.46]. The lowest gap, BG_1_, extends below 500 Hz, achieving deep-subwavelength sound shielding, while the highest gap, BG_5_, spans 3.5–3.7 kHz, demonstrating broadband suppression capability.

Overall, the third-order design attains a total bandgap ratio of 41%, surpassing the 17% and 25% of the first-order and second-order structures and doubling the bandgap density. This indicates that self-similar fractal iteration can systematically enhance sound insulation without altering the macroscopic dimensions. As the fractal order increases, additional mass–spring–mass lattice systems formed by the central slits induce further bandgap splitting. By fine-tuning the channel width *d* and lattice constant *a*, the positions and widths of the gaps can be “tuned” without changing the overall geometry, offering flexible optimization for applications such as acoustic filters and low-frequency noise barriers. Together, the theoretical and numerical analyses validate the significant potential of the circular-loop fractal acoustic metamaterial for deep-subwavelength, broadband sound insulation and structural compactness.

### 3.2. Effective-Parameter Retrieval and Physical Interpretation

For a plane wave incident normal to a slab of thickness *d*, the complex reflection *R* = ∣*R*∣ e^jϕR^ and transmission coefficients *T* = ∣*T*∣ e^jϕT^ obtained from numerical simulation can be converted into effective medium parameters by the following standard procedure:

Intermediate variable:

(12) $$r=\pm \sqrt{(R^{2}-T^{2}-1)-4T^{2}},X=\frac{1-R^{2}+T^{2}+r}{2T}$$

Complex refractive index:

(13) $$n=\frac{-j\ln X+2\pi m}{k_{0}d},m\in Z$$

Here, the integer *m* is chosen to ensure continuity of *R*e(n) across adjacent frequency points.

Normalized characteristic impedance:

(14) $$\varepsilon =\frac{r}{1-2R+R^{2}-T^{2}}$$

Relative effective mass density and bulk modulus:

(15) $$\rho_{eff}=\rho_{0}\varepsilon n$$

(16) $$B_{eff}=\frac{\rho_{0}c_{0}^{2}\varepsilon }{n}$$

**Figure 4:**
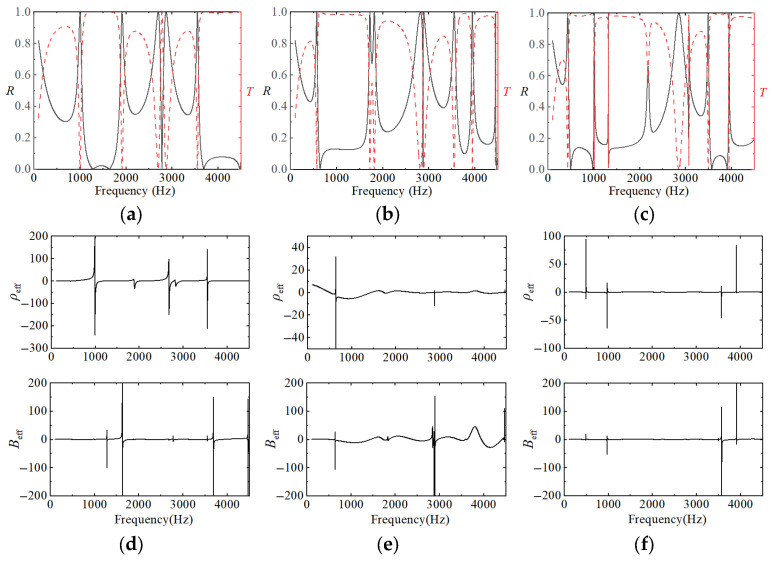
(**a**–**c**) Reflection and transmission coefficients of the fractal acoustic structures: (**a**) the first order, (**b**) the second order, and (**c**) the third order. Analysis results of effective parameters under different fractal structures: (**d**) the first order, (**e**) the second order, and (**f**) the third order.

In the above equations, the simulated band structure results for the first- through third-order fractal acoustic metamaterials—obtained using COMSOL Multiphysics—are shown in Figure 4a–c, and the corresponding retrieved effective parameters are plotted in Figure 4d–f. As the fractal order increases from one to three, both the number of bandgaps and their overall coverage rise markedly. The first-order design supports only two primary bandgaps: within these gaps, transmission is completely suppressed and reflection approaches unity. S-parameter inversion reveals that the first gap is driven by negative effective bulk modulus, whereas the second originates from negative effective density, with the peak amplitude of the retrieved density reaching ±180. Upon doubling the maze path in the second-order structure, the bandgap count increases to five, and the bandgap ratio climbs from ~17% to 27%. In this case, the lowest and third gaps remain dominated by negative bulk modulus, the second and fourth gaps correspond to negative density, and a narrow double-negative window—where both effective density and bulk modulus are simultaneously negative—emerges near 3.6 kHz. At third order, the number of bandgaps further expands to seven, yielding a total coverage of 31%. All identified gaps exhibit concurrent negative density and negative bulk modulus, and the three mid-frequency gaps show alternating sign transitions that steepen the band-edge slopes.

### 3.3. Numerical Validation of Transmission Loss and Sound-Insulation Performance

**Figure 5:**
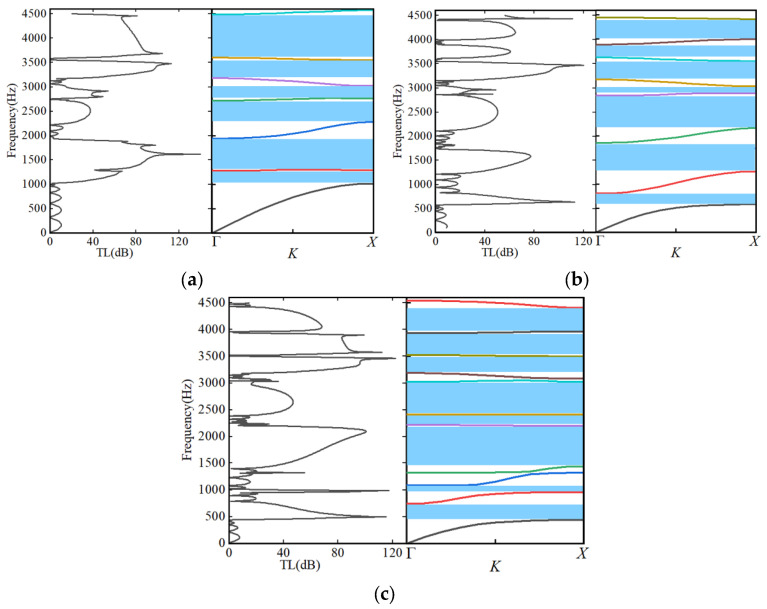
Transmission losses of the fractal acoustic structures with five units: (**a**) the first order, (**b**) the second order, and (**c**) the third order.

Figure 5 comprehensively elucidates the sound-insulation mechanism of the circular-loop fractal acoustic metamaterial through scattering spectra, band structure analysis, and acoustic-pressure fields. After cascading five unit cells along the Γ–X direction: First-order structure (a): Four bandgaps appear only at 1.55–1.75 kHz, 2.35–2.55 kHz, 3.05–3.35 kHz, and 3.6–4.40 kHz, each corresponding to pronounced transmission-loss peaks. Second-order structure (b): Owing to the doubled maze path length, the number of bandgaps increases to seven (the lowest gap extending down to 0.60 kHz). The peak transmission loss is markedly enhanced, and the overall bandgap coverage rises from 47.8% to 52.9%. Third-order structure (c): Eight bandgaps are observed. The first gap’s normalized frequency is as low as 0.043, achieving deep-subwavelength sound insulation. The maximum transmission-loss peak approaches 70 dB, and the bandgap coverage reaches 61.1%.

**Figure 6:**
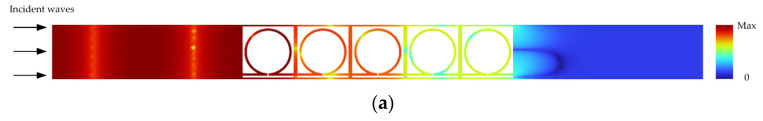

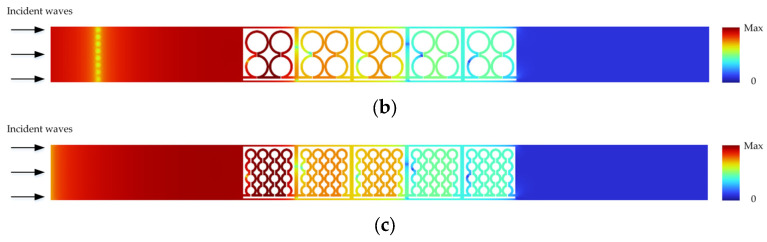
Sound pressure field result: (**a**) the first order at 1620 Hz; (**b**) the second order at 640 Hz; (**c**) the third order at 495 Hz.

The measured sound pressure field distributions are presented in Figure 6. From these data, it can be seen that (a) In the first-order structure at 1620 Hz, the incident wave energy is predominantly reflected and dissipated by the first circular cavity. (b) In the second-order structure at 640 Hz, a checkerboard-like pattern of alternating high- and low-pressure regions emerges. (c) In the third-order structure at 495 Hz, the sixteen nested sub-rings form a dense energy-trapping network, yielding virtually zero transmission at the downstream end of the array. Taken together, increasing the fractal order *n* not only synchronously increases the number and bandwidth of bandgaps and the peak transmission loss but also significantly downshifts the first bandgap—achieving ultra-thin low-frequency broadband sound insulation on the order of *λ*/27. This lightweight, high-performance design offers a compelling solution for machine-room barriers, duct silencers, and traffic noise mitigation.

## 4. Experiment

**Figure 7:**
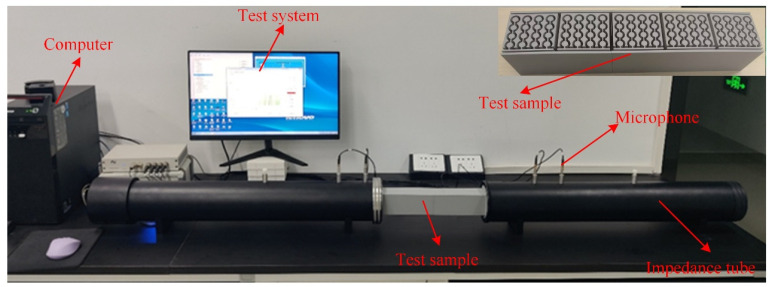
Experimental test bench.

The four-microphone impedance-tube system used in this study comprised three sections: an upstream tube, the sample section, and a downstream tube, as shown in Figure 7. The upstream tube was a rigid duct with an inner diameter of 100 mm and a length of 1000 mm; a 100 W high-power, low-distortion loudspeaker was mounted at its end and driven by a power amplifier to generate a broadband sound source. The downstream tube was approximately 1000 mm long and was terminated with sound-absorbing material to adjust the acoustic impedance. Four BSWA MPA416 microphones (frequency response 20 Hz–20 kHz) captured the sound-pressure signals, which were conditioned by a four-channel preamplifier and then synchronously sampled at 48 kHz using an NI PCIe-6251 data-acquisition card. System control and data logging were implemented on the LabVIEW™ platform.

The test sample was prepared by fused deposition modeling, with a fused deposition layer thickness of 0.1 mm and a nozzle diameter of 0.2 mm. The sample manufacturing consumables are PLA, with a wire diameter of 1.75 mm, and the supplier is Bambu Lab. The geometric dimensions of the test sample are consistent with the simulation. The size of narrow slits of width *d* is 2.5 mm, the size of the lattice constant *a* is 56 mm, and the radius of the ring *R*_3_ is 7 mm.

**Figure 8:**
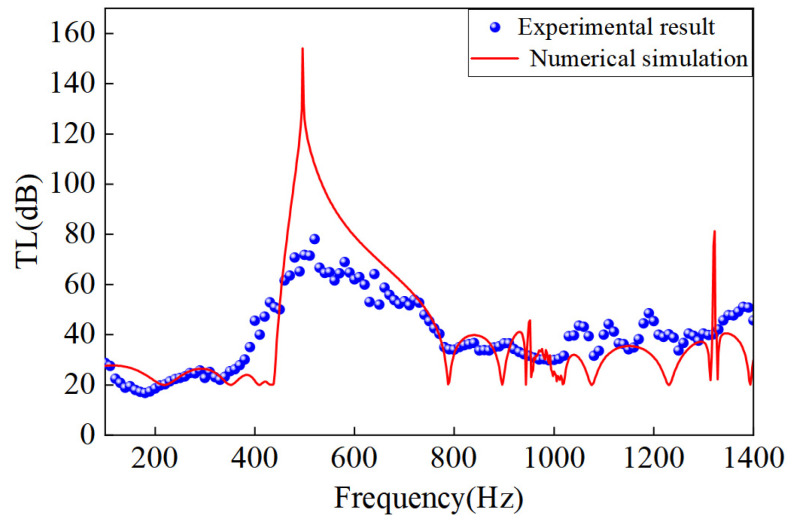
Comparison results of experiments and data simulation.

Transmission-loss (TL) measurements were carried out using the four-microphone transfer-function method in strict accordance with ASTM E2611-17. The time-domain pressure signals recorded at the four microphone positions were first transformed via fast Fourier transform (FFT) to obtain their complex spectra. Knowing each microphone’s location and applying acoustic wave theory, the complex reflection *R* and transmission *T* coefficients of the incident and transmitted waves were computed. The TL spectrum was then calculated pointwise from the ratio of transmitted to incident sound-power levels. The measurement frequency range was set from 100 Hz to 1700 Hz. The entire acquisition, analysis, and TL-curve plotting were automated within the LabVIEW™ environment. The experimentally measured TL is compared against numerical predictions in Figure 8.

As shown in Figure 8, both simulation and experiment exhibit similar features: below 400 Hz, TL remains relatively low; in the 450–700 Hz band, pronounced resonance peaks appear; and above 800 Hz, the TL curve displays pronounced fluctuations. In the numerical model, an extreme TL peak occurs near 580 Hz—corresponding to the lossless cavity-mode resonance predicted by theory. Experimentally, the maximum TL reaches 75 dB, and a broad high-attenuation band spans approximately 520–650 Hz. A slight downward shift of about 10–20 Hz is observed in the experimental peaks relative to the simulation. This shift and the increased bandwidth primarily arise from viscothermal losses at the cavity walls, geometric deviations introduced during sample machining, and nonideal boundary contact between the sample and tube. Overall, these observations indicate that the fractal acoustic metamaterial’s measured performance closely follows the numerical model: the strongest attenuation occurs in the midfrequency band. For practical applications, however, the effects of viscothermal dissipation and fabrication tolerances must be taken into account. Despite the aforementioned quantitative differences, the good agreement in overall trend and midband resonant frequencies (within ~5% error) validates both the design methodology and the reliability of the experimental measurements.

## 5. Conclusions

The circular-loop curve fractal channel acoustic metamaterial introduced in this work demonstrates exceptional low-frequency sound-insulation capabilities. By recursively embedding semicircles and quarter-circles, the fractal-iteration design greatly extends the propagation path compared to conventional maze-type structures, producing multiple low-frequency bandgaps and markedly increasing total bandgap coverage. As the fractal order increases from first to third, the center frequency of the first bandgap shifts dramatically toward lower frequencies, while the overall bandwidth broadens several-fold—revealing outstanding deep-subwavelength attenuation potential. Both numerical transmission-loss simulations and four-microphone impedance-tube measurements validate the superior performance of the third-order design in the few-hundred-Hz range: under ideal, lossless conditions, simulations predict a peak transmission loss of ∼495 Hz, and experiments record a peak of ∼75 dB near the same frequency. Although the experimental peak falls below the theoretical maximum, the observed attenuation trends and bandgap features are in close agreement with predictions. This curve fractal channel metamaterial thus offers a new paradigm for ultrathin, broadband control of low-frequency noise. This article only tested a single fractal element in an impedance tube and has not yet verified its performance in actual scenarios such as large-area sound-absorbing panels or industrial pipelines. The next step will be to design a multi-unit array structure and test its broadband low-frequency sound insulation effect in the reverberation chamber and field applications. And future work will address the influence of material damping and fabrication tolerances and will explore practical integration of these fractal units into engineered noise-control systems.

## Data Availability

The data presented in this study are available upon request from the corresponding author. The raw/processed data needed to reproduce these findings cannot be shared publicly at this time, as they are also part of an ongoing study.
